# The Impact of Palliative Transurethral Resection of the Prostate on the Prognosis of Patients With Bladder Outlet Obstruction and Metastatic Prostate Cancer: A Population-Matched Study

**DOI:** 10.3389/fsurg.2021.726534

**Published:** 2021-10-29

**Authors:** Kun Fang, Pan Song, Jiahe Zhang, Luchen Yang, Peiwen Liu, Ni Lu, Qiang Dong

**Affiliations:** ^1^Department of Urology, West China Hospital of Sichuan University, Chengdu, China; ^2^The Second Clinical Medical College of Lanzhou University, Lanzhou, China; ^3^The First Clinical Medical College of Lanzhou University, Lanzhou, China

**Keywords:** metastatic prostate cancer, bladder outlet obstruction, TURP, survival, SEER

## Abstract

**Objective:** This study aimed to evaluate the survival outcomes of patients with bladder outlet obstruction (BOO) and metastatic prostate cancer (mPCa) after having a palliative transurethral resection of the prostate (pTURP) surgery.

**Methods:** We identified patients with mPCa between 2004 and 2016 in the Surveillance, Epidemiology, and End Results (SEER) database. Patients who received pTURP and non-surgical therapy were identified. A propensity-score matching was introduced to balance the covariate. Kaplan–Meier analysis and COX regression were conducted to evaluate the overall survival (OS) and cancer-specific survival (CSS) outcomes.

**Results:** A total of 36,003 patients were identified; 2,823 of them were in the pTURP group and 33,180 were in the non-surgical group. The survival curves of the overall cohort showed that the pTURP group was associated with worse outcomes in both OS (HR: 1.12, 95% CI: 1.07–1.18, *p* < 0.001) and CSS (HR: 1.08, 95% CI: 1.02–1.15, *p* = 0.004) compared with the non-surgical group. The mean survival time in the overall cohort of the pTURP group was shorter than the non-surgical group in both OS [35.13 ± 1.53 vs. 40.44 ± 0.59 months] and CSS [48.8 ± 1.27 vs. 55.92 ± 0.43 months]. In the matched cohort, the pTURP group had significantly lower survival curves for both OS (HR: 1.25, 95% CI: 1.16–1.35, *p* < 0.001) and CSS (HR: 1.23, 95% CI: 1.12–1.35, *p* < 0.001) than the non-surgical group. pTURP significantly reduced the survival months of the patients (36.49 ± 0.94 vs. 45.52 ± 1.23 months in OS and 50.1 ± 1.49 vs. 61.28 ± 1.74 months in CSS). In the multivariate COX analysis, pTURP increased the risk of overall mortality (HR: 1.19, 95% CI: 1.09–1.31, *p* < 0.001) and cancer-specific mortality CSS (HR: 1.23, 95% CI: 1.14–1.33, *p* < 0.001) compared with the non-surgical group.

**Conclusions:** For mPCa patients with BOO, pTURP could reduce OS and CSS while relieving the obstruction.

## Introduction

Prostate cancer is the most frequently diagnosed non-cutaneous cancer and the second leading cause of cancer-related mortality among men, globally, in 2020 (1, 2). For localized disease, radical prostatectomy (RP) is one of the main treatments with an excellent long-time prognosis (3, 4). However, RP has a low cure rate but a high complication rate for metastatic prostate cancer (mPCa). The majority of patients with mPCa will progress to having metastatic castration-resistant prostate cancer (mCRPC) within 2–3 years despite undergoing intensive androgen deprivation therapy (ADT) (5). Although novel androgen biosynthesis inhibitors such as abiraterone and enzalutamide have been introduced to prolong survival in mCRPC, most patients respond temporarily and soon develop resistance to the inhibitors, which results in the failure to control the tumor progression at the end (6, 7). Hence once the disease enters a castration-resistant state, the patients are incurable by medicine along with therapy and under a substantially greater risk of mortality (1). According to previous literature, about 83.3% of patients with PCa also have bladder outlet obstruction (BOO) (1, 8). Some of these patients have obvious lower urinary tract symptoms (LUTS) and some complications like persistent hematuria, urinary retention, high residual urine volume, bladder stones, etc. These symptoms have been increasingly troublesome for the patients and seriously affect their daily lives.

Patients with BOO who are medicine-failed or have absolute surgical indications are suggested to receive operation intervention, referring to the guidelines (9, 10). The transurethral resection of the prostate (TURP) has been the standard surgical treatment for benign prostatic hyperplasia (BPH) for many years (11). For patients with mPCa with serious BOO who have failed medical therapy and are unwilling to use a catheter for a long time, palliative TURP (pTURP) serves as an effective surgical choice to relieve BOO and improve symptoms (12, 13). However, it creates a dilemma for urologists because it might potentially accelerate the tumor progression. Although several studies have investigated the effects of pTURP on mPCa (14–16), the long-term oncological data is still missing. In this study, we aim to evaluate the prognostic impact of pTURP on patients with mPCa.

## Materials and Methods

### Data Source

The data of this study were extracted from the Surveillance, Epidemiology, and End Results (SEER) database from January 1, 2004, to December 31, 2016. Patients with mPCa were retrospectively identified within the SEER^*^ STAT software. The general information and tumor information were collected.

### Inclusion and Exclusion Criteria

Patients were considered eligible if they met the following criteria: (1) Patients were diagnosed with primary prostate cancer. (2) Prostate cancer was in the metastatic stage (T1-4N0-1M1). (3) Patients received pTURP or non-surgical treatments.

The following criteria were used for data exclusion: (1) Multiple tumors; (2) Patients received other surgical treatments besides pTURP; (3) Important information such as M stages, survival time, and survival status were incomplete or missing.

### Variables and Main Outcomes

We collected the basic characteristics of the patients from the database. The variables involved age, year of diagnosis, race, marital status, tumor, nodes, and metastases (TNM) stage, tumor, prostate-specific antigen (PSA) level, Gleason score, metastasis sites. The main outcomes were cancer-specific survival (CSS) and overall survival (OS).

### Statistical Analysis

A Chi-square test was adopted to assess the differences in the basic characteristics of the pTURP and non-surgical groups. Propensity-score matching was conducted to balance the covariates and generate a new cohort. The survival curves, mean survival months, and 1-, 2-, 3-, 5-, and 10- year survival rates were attained using the Kaplan–Meier analysis. Multivariate COX analyses were performed to evaluate the risk factors. The degrees of risk were presented by the hazard ratio (HR) with a 95% confidence interval (95% CI). *P* < 0.05 was defined as statistically significant. All analyses above were performed with the software SPSS 25 (IBM, Armonk, New York, United States) and Graphed Prism 7.0 (GraphPad Software Inc. San Diego, California, United States).

## Results

### Patient Characteristics

A total of 36,003 patients with mPCa were identified; 2,823 of them received pTURP and 33,180 received non-surgical treatments. The median age was 72 (64–81) years old. After propensity-score matching, 1,942 pairs of patients were matched in the pTURP and non-surgical groups. There were no significant differences in the baseline characteristics between the two groups. The median age was 74 (65–82) and 72 (63–81) years in the matched pTURP and non-surgical groups in the matched cohort. The baseline characteristics of the patients in the overall cohort and matched cohort are presented in Table 1.

**Table 1:** Baseline characteristics of included patients.

**Characteristic**	**Overall cohort**				**Matched cohort**			
	**Total**	**pTURP**	**Non-surgical**	***p***	**Total**	**pTURP**	**Non-surgical**	***p***
**N**	36,003	2,823	33,180		3,884	1,942	1,942	
**Age (years)**								
Median (IQR)	72 (64–81)	49 (47–50)	65 (65–65)		73 (65–81)	74 (65–82)	72 (63–81)	
**Age**, ***n*** **(%)**								
<60 years	6,094 (16.9)	336 (11.9)	5,758 (17.4)	<0.001	454 (11.7)	225 (11.6)	229 (11.8)	0.972
60–69 years	9,892 (27.5)	721 (25.5)	9,171 (27.6)		1,035 (26.6)	514 (26.5)	521 (26.8)	
70–79 years	10,238 (28.4)	882 (31.2)	9,356 (28.2)		1,254 (32.3)	626 (32.2)	628 (32.3)	
≥80 years	9,779 (27.2)	884 (31.3)	8,895 (26.8)		1,141 (29.4)	577 (29.7)	564 (29)	
**Year of diagnosis**, ***n*** **(%)**							
2004–2008	11,600 (32.2)	932 (33)	10,668 (32.2)	0.233	1,428 (36.8)	707 (36.4)	721 (37.1)	0.897
2009–2012	10,577 (29.4)	790 (28)	9,787 (29.5)		1,032 (26.6)	519 (26.7)	513 (26.4)	
2013–2016	13,826 (38.4)	1,101 (39)	12,725 (38.4)		1,424 (36.7)	716 (36.9)	708 (36.5)	
**Race**, ***n*** **(%)**								
White	27,195 (75.5)	2,227 (78.9)	24,968 (75.3)	<0.001	3,161 (81.4)	1,597 (82.2)	1,564 (80.5)	0.372
Black	6,324 (17.6)	390 (13.8)	5,934 (17.9)		518 (13.3)	245 (12.6)	273 (14.1)	
Others	2,484 (6.9)	206 (7.3)	2,278 (6.9)		205 (5.3)	100 (5.1)	105 (5.4)	
**Marriage**, ***n*** **(%)**								
Married	19,956 (55.4)	1,603 (56.8)	18,353 (55.3)	<0.001	2,385 (61.4)	1,192 (61.4)	1,193 (61.4)	0.413
Unmarried	5,783 (16.1)	428 (15.2)	5,355 (16.1)		513 (13.2)	266 (13.7)	247 (12.7)	
Separated	7,790 (21.6)	666 (23.6)	7,124 (21.5)		819 (21.1)	410 (21.1)	409 (21.1)	
Unclear	2,474 (6.9)	126 (4.5)	2,348 (7.1)		167 (4.3)	74 (3.8)	93 (4.8)	
**T stage**, ***n*** **(%)**								
T1–2	17,192 (47.8)	1,616 (57.2)	15,576 (46.9)	<0.001	2,470 (63.6)	1,204 (62)	1,266 (65.2)	0.064
T3–4	7,242 (20.1)	923 (32.7)	6,319 (19)		1,011 (26)	537 (27.7)	474 (24.4)	
Unclear	11,569 (32.1)	284 (10.1)	11,285 (34)		403 (10.4)	201 (10.4)	202 (10.4)	
**N**, ***n*** **(%)**								
N0	17,213 (47.8)	1,612 (57.1)	15,601 (47)	<0.001	2,273 (58.5)	1,147 (59.1)	1,126 (58)	0.771
N1	8,258 (22.9)	635 (22.5)	7,623 (23)		810 (20.9)	402 (20.7)	408 (21)	
Unclear	10,532 (29.3)	576 (20.4)	9,956 (30)		801 (20.6)	393 (20.2)	408 (21)	
**M**, ***n*** **(%)**								
M1a	1,920 (5.3)	139 (4.9)	1,781 (5.4)	0.595	176 (4.5)	79 (4.1)	97 (5)	0.572
M1b	24,677 (68.5)	1,939 (68.7)	22,738 (68.5)		2,997 (77.2)	1,509 (77.7)	1,488 (76.6)	
M1c	7,618 (21.2)	594 (21.0)	7,024 (21.2)		596 (15.3)	297 (15.3)	299 (15.4)	
M1,NOS	1,788 (5.0)	151 (5.3)	1,637 (4.9)		115 (3)	57 (2.9)	58 (3)	
**Tumor sizes**, ***n*** **(%)**							
<1 cm	392 (1.1)	37 (1.3)	355 (1.1)	0.005	30 (0.8)	13 (0.7)	17 (0.9)	0.872
1–2 cm	251 (0.7)	22 (0.8)	229 (0.7)		12 (0.3)	5 (0.3)	7 (0.4)	
2–4 cm	277 (0.8)	23 (0.8)	254 (0.8)		12 (0.3)	7 (0.4)	5 (0.3)	
>4 cm	515 (1.4)	62 (2.2)	453 (1.4)		35 (0.9)	17 (0.9)	18 (0.9)	
Unclear	34,568 (96)	2,679 (94.9)	31,889 (96.1)		3,795 (97.7)	1,900 (97.8)	1,895 (97.6)	
**PSA**, ***n*** **(%)**								
<20.0 ng/ml	5,854 (16.3)	594 (21)	5,260 (15.9)	<0.001	799 (20.6)	416 (21.4)	383 (19.7)	0.428
20.0–97.9 ng/ml	8,216 (22.8)	694 (24.6)	7,522 (22.7)		1,042 (26.8)	518 (26.7)	524 (27)	
≥98.0 ng/ml	17,077 (47.4)	998 (35.4)	16,079 (48.5)		1,530 (39.4)	745 (38.4)	785 (40.4)	
Unclear	4,856 (13.5)	537 (19)	4,319 (13)		513 (13.2)	263 (13.5)	250 (12.9)	
**Gleason score**, ***n*** **(%)**							
≤ 7	4,679 (13)	409 (14.5)	4,270 (12.9)	<0.001	534 (13.7)	243 (12.5)	291 (15)	0.077
8	17,170 (47.7)	2,141 (75.8)	15,029 (45.3)		2,939 (75.7)	1,494 (76.9)	1,445 (74.4)	
9–10	14,154 (39.3)	273 (9.7)	13,881 (41.8)		411 (10.6)	205 (10.6)	206 (10.6)	
**Bone metastases**, ***n*** **(%)**							
Yes	19,262 (53.5)	1,473 (52.2)	17,789 (53.6)	0.142	2,032 (52.3)	1,028 (52.9)	1,004 (51.7)	0.441
No/unclear	16,741 (46.5)	1,350 (47.8)	15,391 (46.4)		1,852 (47.7)	914 (47.1)	938 (48.3)	
**Brain metastases**, ***n*** **(%)**							
Yes	254 (0.7)	8 (0.3)	246 (0.7)	0.005	11 (0.3)	4 (0.2)	7 (0.4)	0.365
No/unclear	35,749 (99.3)	2,815 (99.7)	32,934 (99.3)		3,873 (99.7)	1,938 (99.8)	1,935 (99.6)	
**Liver metastases**, ***n*** **(%)**							
Yes	1,006 (2.8)	123 (4.4)	883 (2.7)	<0.001	48 (1.2)	22 (1.1)	26 (1.3)	0.561
No/unclear	34,997 (97.2)	2,700 (95.6)	32,297 (97.3)		3,836 (98.8)	1,920 (98.9)	1,916 (98.7)	
**Lung metastases**, ***n*** **(%)**							
Yes	1,784 (5)	148 (5.2)	1,636 (4.9)	0.463	98 (2.5)	46 (2.4)	52 (2.7)	0.539
No/unclear	34,219 (95)	2,675 (94.8)	31,544 (95.1)		3,786 (97.5)	1,896 (97.6)	1,890 (97.3)	
**Radiotherapy**								
Yes	7,742 (21.5)	487 (17.3)	7,255 (21.9)	<0.001	639 (16.5)	315 (16.2)	324 (16.7)	0.729
No/unclear	28,261 (78.5)	2,336 (82.7)	25,925 (78.1)		3,245 (83.5)	1,627 (83.8)	1,618 (83.3)	
**Chemotherapy**								
Yes	3,665 (10.2)	290 (10.3)	3,375 (10.2)	0.865	286 (7.4)	145 (7.5)	141 (7.3)	0.806
No/unclear	32,338 (89.8)	2,533 (89.7)	29,805 (89.8)		3,598 (92.6)	1,797 (92.5)	1,801 (92.7)	

### Survival Curves

**Figure 1:**
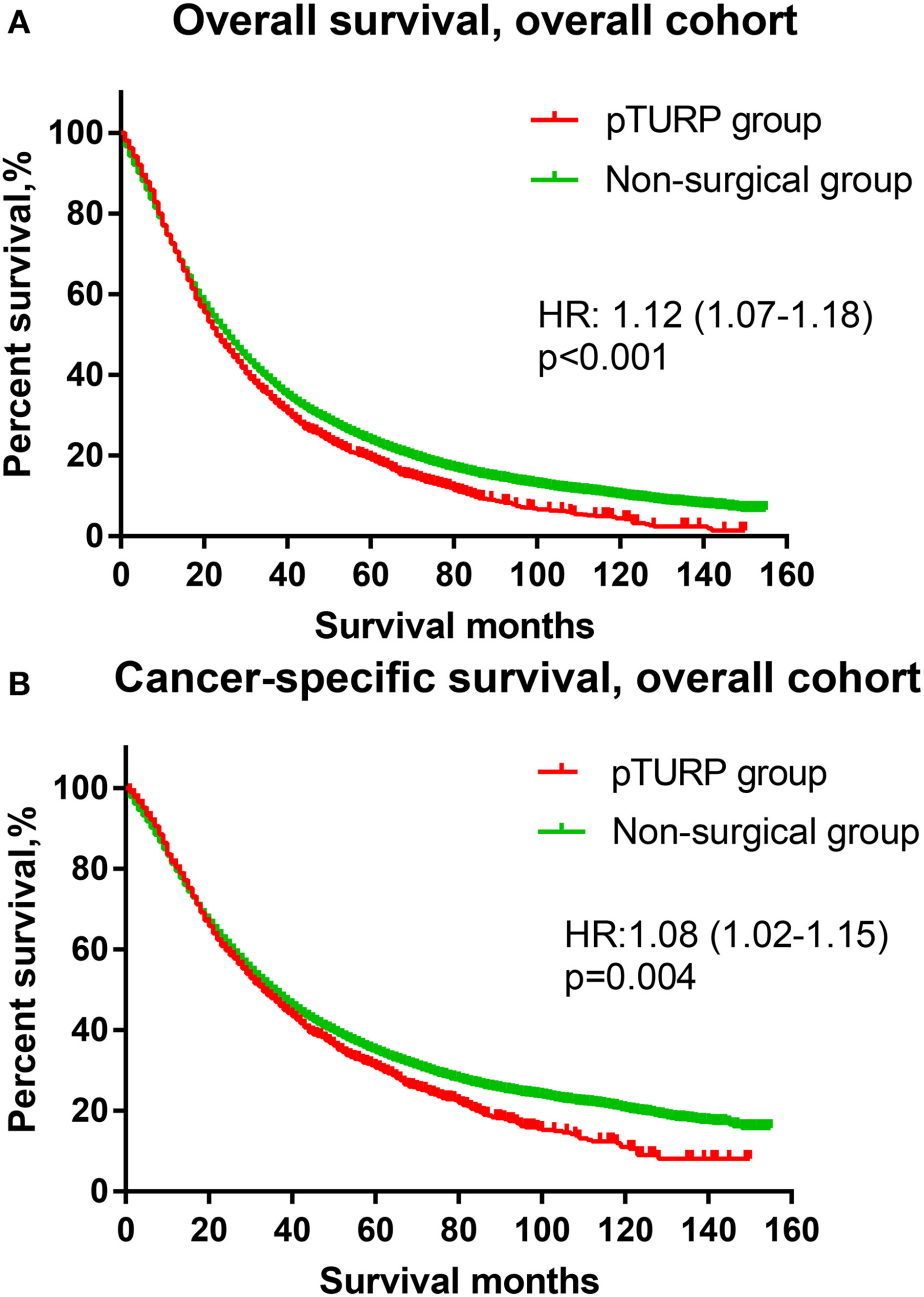
The overall survival (OS) and cancer-specific survival (CSS) curves of the transurethral resection of the prostate (TURP) and non-surgical group for patients with metastatic prostate cancer (mPCa) in the overall cohort. **(A)** OS curve. **(B)** CSS curve.

**Figure 2:**
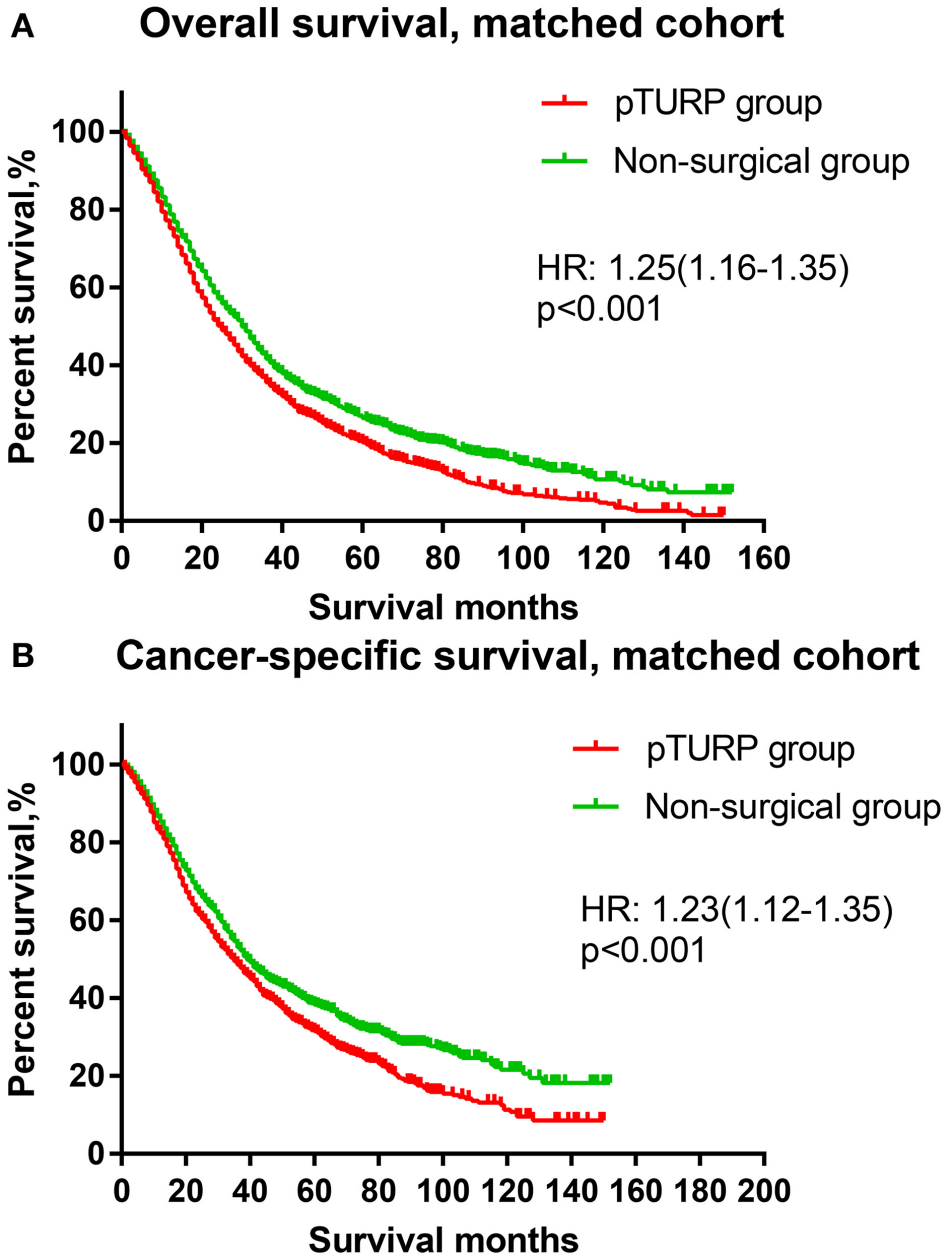
The OS and CSS curves of the TURP and non-surgical group for patients with mPCa in the matched cohort. **(A)** OS curve **(B)** CSS curve.

The OS curve of the overall cohort revealed that the pTURP group was associated with worse long-term survival outcomes than the non-surgical group (HR: 1.08, 95% CI: 1.02–1.15, *p* = 0.004, Figure 1A). As for the CSS curve of the overall cohort, the pTURP group also showed worse survival outcomes than the non-surgical group (HR: 1.08, 95% CI: 1.02–1.15, *p* = 0.004, Figure 1B). In the matched cohort, the pTURP group was associated with worse outcomes than the non-surgical group in both OS (HR: 1.25, 95% CI: 1.16–1.35, *p* < 0.001, Figure 2A) and CSS (HR: 1.23, 95% CI: 1.12–1.35, *p* < 0.001, Figure 2B).

### Survival Time and Survival Rates

In the overall cohort, the 1-year survival rates of the pTURP and non-surgical group were 71.6 vs. 69.1% in OS and 79.7 vs. 76.9% in CSS. The pTURP group was associated with similar 2- and 3-year CSS (59.2 vs. 59.7% in 2-year and 46.6 vs. 47.5% in 3-year) and worse OS (47.5 vs. 49.3% in 2-year and 33.5 vs. 36.4% in 3-year) than the non-surgical group. Comparing the 5-year survival rates, the pTURP group was associated with significantly worse outcomes than the non-surgical group in both OS (18.7 vs. 22.3%) and CSS (30.4 vs. 33.4%). For the mean survival time, that of the pTURP group was significantly shorter than the non-surgical group in both OS (35.13 ± 1.53 vs. 40.44 ± 0.59 months) and CSS (48.8 ± 1.27 vs. 55.92 ± 0.43 months). These results are presented in Table 2.

**Table 2:** The survival rates and survival time in the TURP group and non-surgical group in the overall cohort and matched cohort.

**Variables**	**OS**		**CSS**	
	**pTURP group**	**Non-surgical group**	**pTURP group**	**Non-surgical group**
**Survival rates in overall cohort,%**				
1-year OS	71.6% (69.8–73.4%)	69.1% (68.5–69.7%)	79.7% (78.1–81.3%)	76.9% (76.5–77.3%)
2-year OS	47.5% (45.5–49.5%)	49.3% (48.7–49.9%)	59.2% (57–61.4%)	59.7% (59.1–60.3%)
3-year OS	33.5% (31.5–35.5%)	36.4% (35.8–37%)	46.6% (44.2–49%)	47.5% (46.9–48.1%)
5-year OS	18.7% (16.9–20.5%)	22.3% (21.7–22.9%)	30.4% (27.9–32.9%)	33.4% (32.6–34.2%)
10-year OS	4.4% (3.0–5.8%)	9.4% (8.8–10%)	10.9% (8.0–13.8%)	19.6% (18.8–20.4%)
**Survival time in overall cohort, months**				
Mean ± SD	35.13 ± 1.53	40.44 ± 0.59	48.8 ± 1.27	55.92 ± 0.43
**Survival rates in matched cohort,%**				
1-year OS	74.2% (72.2–76.2%)	77.1% (75.1–79.1%)	81.8% (80–83.6%)	84% (82.2–85.8%)
2-year OS	49.5% (47.1–51.9%)	55.9% (53.5–58.3%)	61% (58.5–63.5%)	66.1% (63.7–68.5%)
3-year OS	35.1% (32.7–37.5%)	40.7% (38.3–43.1%)	48.2% (45.5–50.9%)	52.3% (49.8–54.8%)
5-year OS	19.9% (17.7–22.1%)	25.9% (23.5–28.3%)	31.2% (28.3–34.1%)	33.7% (30.8–36.6%)
10-year OS	4.6% (3.0–6.2%)	10.5% (8.1–12.9%)	11.3% (8.0–14.6%)	21.3% (17.4–25.2%)
**Survival time in matched cohort, months**				
Mean ± SD	36.49 ± 0.94	45.52 ± 1.23	50.1 ± 1.49	61.28 ± 1.74

In the matched cohort, the OS and CSS of the pTURP group were consistently worse than the non-surgical group. Among the 1-, 3-, and 5-year survival rates, the CSS of the pTURP and non-surgical group were 81.8 vs. 84%, 48.2 vs. 52.3%, and 31.2 vs. 33.7%, respectively. The 1-, 3-, and 5- year OS rates of the pTURP and non-surgical group were 74.2 vs. 77.1%, 49.5 vs. 55.9%, 35.1 vs. 40.7%, 19.9 vs. 25.9%, and 4.6 vs. 10.5%, respectively. The mean survival time of the pTURP group was shorter than that of the non-surgical group in OS (36.49 ± 0.94 vs. 45.52 ± 1.23 month) and CSS (50.1 ± 1.49 vs. 61.28 ± 1.74 months). These results are also shown in Table 2.

### Multivariate COX Analysis for OS and PCSS

The results of the COX analyses were showed in Table 3. With the non-surgical therapy group as the reference, the HR and 95% CI of the pTURP group for both OS and CSS were 1.19 (1.09–1.31) and 1.23 (1.14–1.33) individually. With the M1a stage as the reference, the HR and 95% CI of the CSS of M1b and M1c were 1.89 (1.44–2.49) and 2.19 (1.64–2.92), respectively. With the patients without metastases as the reference, the CSS HR and 95% CI of the patients with bone metastases, lung metastases, liver metastases, and brain metastases were 1.09 (1.04–1.14), 1.08 (1–1.17), 1.51 (1.28–1.79), and 2.29 (2.1–2.5), respectively.

**Table 3:** Multivariate COX analysis for patients in the matched cohorts.

**Risk factors**	**OS**		**CSS**	
	**HR (95% CI)**	***p***	**HR (95% CI)**	***p***
**Age**				
<60 years	1	Ref.	1	Ref.
60–69 years	0.93 (0.81–1.07)	0.306	0.84 (0.72–0.98)	0.023
70–79 years	1.15 (1–1.32)	0.045	0.92 (0.79–1.07)	0.275
≥80 years	1.72 (1.49–1.98)	<0.001	1.16 (0.99–1.36)	0.074
**Year of diagnosis**			
2004–2008	1	Ref.	1	Ref.
2009–2012	0.85 (0.74–0.98)	0.025	0.92 (0.79–1.08)	0.321
2013–2016	0.82 (0.69–0.98)	0.025	0.86 (0.7–1.05)	0.139
**Race**				
White	1	Ref.	1	Ref.
Black	1 (0.89–1.12)	0.956	0.97 (0.84–1.11)	0.648
Others	0.78 (0.65–0.94)	0.007	0.79 (0.63–0.97)	0.027
**Marital status**				
Married	1	Ref.	1	Ref.
Unmarried	1.15 (1.01–1.3)	0.036	1.12 (0.97–1.29)	0.141
Separated	1.21 (1.1–1.33)	<0.001	1.23 (1.1–1.37)	<0.001
**T stage**				
T1–2	1	Ref.	1	Ref.
T3–4	1.16 (1.06–1.27)	0.001	1.18 (1.06–1.31)	0.003
**N**				
N0	1	Ref.	1	Ref.
N1	1.14 (1.02–1.26)	0.02	1.2 (1.06–1.36)	0.004
**M**				
M1a	1	Ref.	1	Ref.
M1b	1.53 (1.22–1.91)	<0.001	1.89 (1.44–2.49)	<0.001
M1c	1.85 (1.46–2.34)	<0.001	2.19 (1.64–2.92)	<0.001
**PSA**				
<20.0 ng/ml	1	Ref.	1	Ref.
20.0–97.9 ng/ml	1.01 (0.9–1.13)	0.938	0.98 (0.86–1.13)	0.805
≥98.0 ng/ml	1.13 (1.02–1.26)	0.025	1.18 (1.04–1.34)	0.011
**Gleason score**				
≤ 7	1	Ref.	1	Ref.
8	1.42 (1.26–1.59)	<0.001	1.48 (1.29–1.7)	<0.001
9–10	1.81 (1.53–2.14)	<0.001	1.8 (1.47–2.2)	<0.001
**Bone metastases**			
Yes	1	Ref.	1	Ref.
No/unclear	0.97 (0.84–1.13)	0.7	1.07 (0.9–1.26)	0.469
**Brain metastases**			
Yes	1	Ref.	1	Ref.
No/unclear	0.47 (0.24–0.92)	0.027	0.39 (0.19–0.79)	0.009
**Liver metastases**			
Yes	1	Ref.	1	Ref.
No/unclear	0.35 (0.26–0.49)	<0.001	0.29 (0.2–0.41)	<0.001
**Lung metastases**			
Yes	1	Ref.	1	Ref.
No/unclear	1.19 (0.89–1.59)	0.243	1.09 (0.78–1.51)	0.617
**Surgery**				
pTURP	1	Ref.	1	Ref.
Non-surgical therapy	1.19 (1.09–1.31)	<0.001	1.23 (1.14–1.33)	<0.001

## Discussion

Most of the patients with the metastatic prostate disease will develop castration resistance, which means that they will face an unfavorable prognosis and survival (17). For patients with severe BOO and mPCa, whether or not to perform pTURP is a dilemma. Its benefits and harms need to be balanced for these patients. pTURP can relieve obstruction, improve lower urinary tract symptoms and the complications of BOO, and damage the primary tumor. But it may create a source for new metastasis like residual tumor and cancerous cell debris. The channel opened by pTURP operation may potentially promote the spread of tumor cells or tumor-promoting growth factors *via* the vascular passage (18). Additionally, the necessity of pTURP might imply a much severe condition of PCa, associated with hazardous complications such as a larger size of the tumor and a more aggressive or quick-spreading tumor (14). Poorly differentiated or aggressive prostate tumors are more likely to lead to invasive microvessel density and irregular vessel lumen (18–20).

Our results found that pTURP could significantly reduce the OS and CSS outcomes in both the overall and matched cohort. The mean survival time of the pTURP group was 36.49 vs. 45.52 months in OS and 50.1 vs. 61.28 months in CSS, compared with the non-surgical group in the matched cohort. This finding was consistent with several former retrospective studies. Choi et al. (14) reviewed 614 patients who received ADT, and pTURP showed notably lower survival rates among castration-resistant prostate cancer (CRPC)-free survival, OS, and CSS than the ADT only group. They specially regarded pTURP as an independent hazard factor of CSS with an HR and 95% CI of 2.543 (1.008–6.420). Pelletier et al. (21) found that the 5-year OS of patients after pTURP was only 16% (95% CI: 6.5–29.8). It was similar to the 18.7% of our 5-year OS results. Jin et al. (22) reported pTURP as an independent risk factor for the biochemical recurrence for prostate cancer, leading to significantly lower survival rates. Krupski et al. (16) also concluded that pTURP was an adverse prognostic factor even after the adjustment of the classical tumor characteristics. Note that studies also demonstrated that generally, the pTURP group had a larger prostate volume than the non-pTURP group before matching, which may contribute to the unfavorable prognosis in pTURP. Interestingly, the resection weight, resection time, and blood transfusion were tested not correlated with CSS (14, 22).

There were also some opposite opinions. Qin et al. (23) analyzed the curative effect of complete androgen blockade (CAB) therapy only and pTURP+CAB combined therapy for patients with metastatic hormone sensitive prostate cancer (mHSPC). Their study revealed that pTURP was beneficial to both the OS (24.4 vs. 22.9 months) and CSS (24.4 vs. 24.1 months), resulting in a more prolonged and sensitive response to hormone therapy in mHSPC. Qu et al. (24) included 118 patients with mPCa; 110 of them were in the pTURP+ADT group. They reported that the 3-year CSS of pTURP + ADT group was higher than that in the ADT alone group (95.9 vs. 64.9%, *p* = 0.004). In their analysis, it was found that pTURP + ADT could improve the CSS outcomes when PSA ≥65 ng/mL, Gleason Score (GS) ≥8, and bone metastasis ≤ 5. As expected, pTURP could obviously improve LUTS symptoms in these patients. Crain et al. (12) reported that pTURP could be performed safely with significant improvement in the urinary symptoms and quality of life in mPCa patients. Moreover, Sehgal et al. (20) revealed that pTURP is a necessity for patients with PCa and high Gleason sum and the presence of retention, which showed an ideal 6-month catheter free rate of 72%. Therefore, the application of pTURP in patients with mPCa and serious BOO should be comprehensively considered.

Even though our study analyzed a large sample of patients with long-time follow-ups, certain limitations still existed in our study. The limitations were as follows: (1) for patients with mPCa, medical treatments like ADT were essential factors for the control of disease progression. However, limited by the original data from the SEER database, this information was unavailable and cannot be obtained. We were unable to identify the patients with the same initial treatment, for example, patients who received enzulatamide + pTURP vs. enzulatamide alone. It is the biggest limitation of our study. (2) Limited by the raw data in the database, the information of BOO and the intraoperative information such as prostate volume, operative time, blood loss, and postoperative complications were unavailable. This information might have a relation with OS. It might cause interferences for the results. (3) Only the survival outcomes were analyzed in our study. Many important outcomes such as biochemical recurrence-free survival, urinary symptoms, and quality of life, were not analyzed because of the raw data in the database. (4) For pTURP, some new minimally invasive methods, such as laser vaporization therapy featuring high-energy vaporization of prostate tissue, may have a better effect in confining tumor spread. Further analysis is needed to eliminate the deficiency of these methods on mPCa patients.

## Conclusion

For patients with mPCa and BOO, pTURP could reduce OS and CSS to a certain degree while relieving the obstruction. However, with the limitation of our study, more high-quality studies are needed for further evaluation.

## Data Availability Statement

The datasets presented in this study can be found in online repositories. The names of the repository/repositories and accession number(s) can be found in the article/supplementary material.

## Author Contributions

KF, PS, and QD designed the protocol, extracted and analyzed the data, drafted the manuscript, revised the manuscript, approved the final manuscript, and supervised all stages of this study. JZ, LY, PL, and NL extracted and analyzed the relevant data, drafted the manuscript, revised the manuscript, and approved the final manuscript. All authors contributed to the article and approved the submitted version.

## Funding

This work was supported by a Key Project of National Natural Science Foundation of China; Grant ID: 8177060452; and 1.3.5 project for disciplines of excellence, West China Hospital, Sichuan University, Grant ID: ZY2016104.

## Conflict of Interest

The authors declare that the research was conducted in the absence of any commercial or financial relationships that could be construed as a potential conflict of interest.

## Publisher's Note

All claims expressed in this article are solely those of the authors and do not necessarily represent those of their affiliated organizations, or those of the publisher, the editors and the reviewers. Any product that may be evaluated in this article, or claim that may be made by its manufacturer, is not guaranteed or endorsed by the publisher.
